# Antimicrobial resistance of *Escherichia coli* isolates from cattle in Eastern Algeria

**DOI:** 10.14202/vetworld.2019.1195-1203

**Published:** 2019-08-07

**Authors:** Djanette Barour, Amine Berghiche, Nadji Boulebda

**Affiliations:** 1 Department of Veterinary Science, Institute of Agronomic and Veterinarian Sciences, University of Mohamed Cherif Messaâdia, Souk Ahras, Algeria; 2 Laboratory of Science and Technique of the Living, University of Mohamed Cherif Messaâdia, Souk Ahras, Algeria

**Keywords:** antimicrobial resistance, cattle, Eastern Algeria, *Escherichia coli*

## Abstract

**Background and Aim:**

Lack of information about the antibiotic resistance in commensal *Escherichia coli* from Algerian livestock prompted us to do this study to determine the different levels of antimicrobial susceptibility, antibiotic multidrug resistance (MDR) rates, and phenotypical patterns of *E. coli* strains isolated from healthy cattle to control the spread of animal-resistant strains to humans and the environment.

**Materials and Methods:**

A total of 198 cattle were sampled (swabbed in the rectum), reared in the farms of Souk Ahras, Tebessa, and Oum el Bouaghi governorates of Eastern Algeria. Isolation of *E. coli* strains was performed on MacConkey agar and then the different strains were identified to the species level using an API 20E identification kit. Antimicrobial susceptibility was determined using a panel of 13 antibiotic disks by disk diffusion method on Mueller-Hinton agar. The double-disk synergy test with cefotaxime and amoxicillin-clavulanate disks was used for the screening of extended-spectrum beta-lactamase phenotypes. For colistin susceptibility, the minimum inhibitory concentration was examined using broth microdilutions technique.

**Results:**

The results showed that among the 198 *E. coli* isolates, elevated resistance rates were observed for ampicillin (59.09%) and tetracycline (43.43%), and moderate resistance rates for cephalothin (16.16%), trimethoprim/sulfamethoxazole (15.15%), and amoxicillin/clavulanate (11.62%); however, low resistance rates were found for nalidixic acid (8.08%), ciprofloxacin (7.07%), kanamycin (6.56%), cefotaxime (4.54%), chloramphenicol (4.04%), nitrofurantoin (2.52%), cefoxitin (2.02%), gentamycin (1.01%), and no resistance to colistin. However, nine extended-spectrum ß-lactamases producing *E. coli* strains were identified. Forty-four different patterns were determined, indicating a wide variety of resistance, ranging from one antimicrobial to a combination of 10. Analysis of coresistances revealed that 63 isolates (31.82%) were susceptible to all antibiotics used in the study, 42 isolates (21.21%) were resistant to one antibiotic, 43 isolates (21.72%) were resistant to two antibiotics, 24 isolates (12.12%) resistant to three antibiotics, 26 isolates (13.13%) were resistant for more than three agents, and 45 isolates (22.73%) were MDR (which means resistant to three or more families of antibiotics).

**Conclusion:**

This study demonstrates that commensal *E. coli* remains a potential source of antibiotic resistance in view of the high prevalence of antimicrobial resistance. The vast range of MDR phenotypes, especially extended-spectrum ß-lactamases producing strains, emphasizes the urgent requirement to adopt measures to control the use of antimicrobials, in particular, by private veterinarians, as well as the strengthening of veterinary surveillance networks for antimicrobial resistance to control the spread of MDR bacteria from animals to humans and the environment.

## Introduction

Antimicrobial agents are used therapeutically in animals and humans for control of bacterial infections and may be incorporated into commercial livestock and poultry feed at subtherapeutic doses for growth promotion [1,2]. Antibiotics have significantly reduced mortality associated with infectious diseases during the 20^th^ century; unfortunately, their massive and repeated use, in animal farming, has led to the emergence of bacteria multidrug resistance (MDR) to these drugs [3]. While antimicrobials are used to target pathogenic organisms, simultaneous selection pressure is exerted on the commensal enterobacteria, encouraging the development and maintenance of antimicrobial resistance in these bacteria [4]. Both antimicrobial-resistant pathogens and commensal organisms can disseminate to humans through direct contact with animals [5] or through the food chain [6,7]. Despite the abundance of phenotypes of antibiotic resistance observed within bacteria, only four mechanisms by which these resistances are acquired have been described, all of which are controlled by the action of specific genes: Enzymatic inactivation or modification of antimicrobial agents, impermeability of the bacteria cell wall or membrane, active expulsion of the drug by the cell efflux pump, and alteration in target receptors [8,9]. Thus, the genes coding for the antibacterial resistance determinants are located either on the bacterial chromosome, or on mobile genetic elements such as plasmids, transposons, and integrons and can be transmitted vertically and horizontally [8,10].

Commensal *Escherichia coli* are part of the intestinal flora of human and animals with certain strains being pathogenic and causing conditions including gastroenteritis, cystitis, meningitis, peritonitis, and septicemia. Changes in the antibiotic resistance of this species may serve as an early warning of the development of resistance by related pathogenic bacteria [7,11-13]. Several international studies have been reported on the fecal carriage of resistant *E. coli* in cattle [14-18]; but in Algeria, the majority of published papers are focused only on human pathogenic strains of *E. coli* [19-22], while some papers have been reported on resistant *E. coli* strains in poultry [23,24], the current information on antimicrobial resistance in commensal *E. coli* strains in cattle is very limited.

Lack of information about the antibiotic resistance in commensal *E. coli* from livestock requires us to deepen research in this area to know the epidemiology of antimicrobial susceptibility of *E. coli* strains and contribute for a better use of antibiotics in Algerian cattle, whose food is intended for human consumption. For these reasons, we carried out this study using the disk diffusion method on Mueller-Hinton agar, in *E. coli* isolates from healthy cattle during a period of 2 years, to determine their different levels of antimicrobial susceptibility, the rates of MDR, and their different phenotypically patterns of antibiotic resistance.

## Materials and Methods

### Ethical approval

This study did not require any ethical approval from the University Animal Ethics Committee and was performed in accordance with Algerian laws and regulations on animal welfare.

### Study area

**Figure-1:**
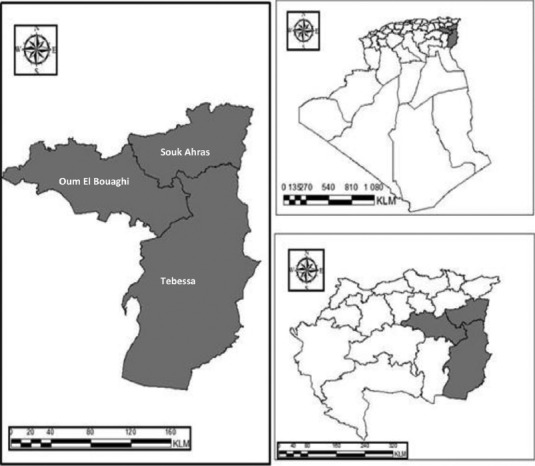
Map of Algeria showing the sampling sites of the study (The map was generated in “ESRI® ArcGIS 9.1 for desktop” software).

This study was carried out in several localities of the governorates of Souk Ahras, Tébessa, and Oum el Bouaghi, all located in the east of Algeria (Figure-1).

### Sample collection

From March 2016 to March 2018, 198 cattle were swabbed in the rectum. Swabs loaded with feces were then dissolved into 2 ml of sterile physiological saline and sent directly to the laboratory.

### *E. coli* isolation and identification

The samples were inoculated on Mac Conkey agar for 24 h at 37°C; positive lactose colonies were tested by Gram stain and oxidase testing. The isolates were then identified to the species level using an API 20E identification kit (bioMérieux, France).

### Antimicrobial susceptibility testing

Antibiotic sensitivity was determined using disk diffusion method on Mueller-Hinton agar (Merck), following Clinical and Laboratory Standards Institute (CLSI) standards [25]. The double-disk synergy test with cefotaxime and amoxicillin-clavulanate disks was used for the screening of extended-spectrum ß-lactamases (ESBL)phenotype. *E. coli* ATCC 25922 and *Klebsiella pneumoniae* ATCC 700603 were used as ESBL negative and positive reference strains, respectively.

The antibiotics tested (Oxoid) their concentrations and the breakpoints are shown in Table-1 [25,26]. Only for colistin susceptibility, the minimum inhibitory concentration (MIC) was examined using broth microdilutions technique (Table-1) [25,26]. The MIC for each isolate was read as the lowest dilution demonstrating no visible growth, based on CLSI [26].

**Table 1:** Disk drug concentrations and diffusion zone breakpoints for antimicrobial sensitivity and the minimal inhibitory concentration only for colistin sensitivity [25,26].

Antimicrobial agent (drug code)	Disk drug concentration (mg)	Breakpoints (mm)		

		Sensitive	Intermediate	Resistant
AMP	10 μg	≥17	14-16	≤13
AMC	20/10 μg	≥18	14-17	≤13
KF	30 μg	≥18	15-17	≤14
CTX	30 μg	≥26	23-25	≤22
Fox	30 μg	≥18	15-17	≤14
k	30 μg	≥18	14-17	≤13
CN	10 μg	≥15	13-14	≤12
SXT	1.25/23.75 μg	≥16	11-15	≤10
Te	30 μg	≥15	12-14	≤11
Na	30 μg	≥19	14-18	≤13
CIP	5 μg	≥21	16-20	≤15
F	300 μg	≥17	15-16	≤14
C	30 μg	≥18	13-17	≤12
CT	MIC (μg/ml)			
	WT		NWT	
	≤2		≥4	

WT=Wild type, NWT=Non-wild type, MIC=Minimum inhibitory concentration , AMP=Ampicillin, AMC=Amoxicillin/clavulanate, KF=Cephalothin, CTX=Cefotaxime, Fox=Cefoxitin, k=Kanamycin, CN=Gentamycin, SXT=Trimethoprim/sulfamethoxazole, Te=Tetracyclines, Na=Nalidixic acid, CIP=Ciprofloxacin, F=Nitrofurantoin, C=Chloramphenicol, CT=Colistin

### Statistical analysis

**Figure-2:**
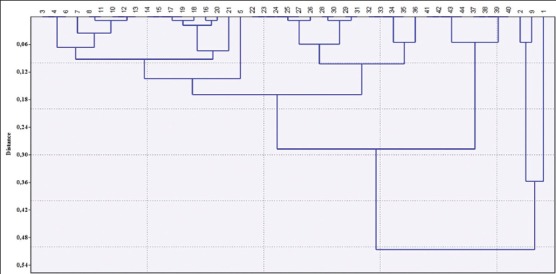
Patterns of antimicrobial resistance phenotypes for *E. coli* strains isolated in the study. The upper margin indicates the antibiogram patterns of phenotypic antimicrobial resistance detected from 1 to 44. The left margin indicates the distance between the different clusters.

- Descriptive analysis: The graphic representation was performed using the program (Microsoft Office Excel, 2007).
- Data analysis: To compare coresistances in different isolates, the Kruskal–Wallis test was used. This test was a non-parametric statistical test that assesses the differences among three or more independently sampled groups on a single, non-normally distributed continuous variable [27].
- Cluster analysis: We used the free software (Past 3.22), to determine the coefficient of correlation between the number of resistances and the number of strains for each of the antibiogram profiles detected, using algorithm paired group and similarity measure using Euclidean distance. The correlation coefficient was equal to 0.07798 (Figure-2).

## Results and Discussion

This study aimed to determine the resistance rates of commensal *E. coli* strains to a panel of 13 antibiotics belonging to nine different families, as well as to determine the rates of MDR and the different phenotypical patterns of antibiotic resistance. Among the 198 fecal samples, 198 *E. coli* strains were isolated, which represents 100% isolation rate; this percentage is in accordance with Bywater *et al*. [28].

### Antibiotic resistance rates

The resistance rates (Table-2) of the 198 isolated *E. coli* show high rates of resistance for ampicillin (AMP) (59.09%) and tetracycline (TE) (43.43%), moderate rates of resistance were observed for cephalothin (16.16%), trimethoprim/sulfamethoxazole (SXT) (15.15%), and amoxicillin/clavulanate (11.62%); however, the low resistance rates have been observed for nalidixic acid (8.08%), ciprofloxacin (7.07%), kanamycin (6.56%), cefotaxime (4.54%), chloramphenicol (4.04%), nitrofurantoin (2.52%), cefoxitin (2.02%), gentamycin (1.01%), and no resistance was recorded for colistin (0%). However, nine extended-spectrum beta-lactamases producing *E. coli* were detected.

**Table 2:** Frequencies of antibiotics resistance in *E. coli* isolates.

Families of antibiotics	Antibiotics	Sensitive isolates	Resistant isolates
	
		n (%)	n (%)
Beta-lactams	AMP	81 (40.91)	117 (59.09)
	AMC	175 (88.38)	23 (11.62)
Cephalosporin	KF	166 (83.84)	32 (16.16)
	CTX	189 (95.45)	9 (4.54)
	FOX	194 (97.98)	4 (2.02)
Aminoglycosides	K	185 (93.43)	13 (6.56)
	CN	196 (98.99)	2 (1.01)
Sulfonamides	SXT	168 (84.85)	30 (15.15)
Cyclins	Te	112 (56.56)	86 (43.43)
Quinolones	NA	182 (91.92)	16 (8.08)
	CIP	184 (92.93)	14 (7.07)
Polymyxins	CT	198 (100)	0 (0)
Nitrofurans	F	193 (97.47)	5 (2.52)
Phenicols	C	190 (95.96)	8 (4.04)

AMP=Ampicillin, AMC=Amoxicillin+clavulanate, KF=Cephalothin, CTX=Cefotaxime, FOX=Cefoxitin, K=Kanamycin, CN=Gentamycin, SXT=Trimethoprim/sulfamethoxazole, Te=Tetracycline, NA=Nalidixic acid, CIP=Ciprofloxacin, CT=Colistin, F=Nitrofurantoin, C=Chloramphenicol

**Figure-3:**
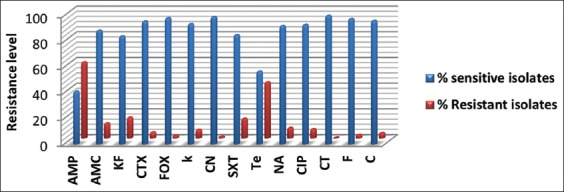
Frequencies of antibiotics resistance in *Escherichia coli* isolates.

Beta-lactams are the most commonly used antibiotics (Figure-3) for the treatment of infections caused by *Enterobacteriaceae*. Resistance to beta-lactam antibiotics develops as a result of mutations or the acquisition of genetic material such as plasmids, transposons, or integrons from other resistant bacteria [29]. In Eastern Algeria, this class of antibiotics is widely used [2,30]. Their extensive and long-term use render their resistance rate high as a function of selection pressure, this resistance in *E. coli* is mostly ensured by ß-lactamases, which presently contains more than 200 enzymes that hydrolyze the ß-lactam cycle and inactivate it in a manner that represents a very high risk to public health [31,32].

Among ß-lactam antibiotics, AMP is one of the oldest drugs used in bovines; it is indicated for the treatment of septicemia, digestive, respiratory, and genitourinary infections [33]; this antimicrobial has the highest resistance rate with 59.09%, the finding is compatible with that of Sawant *et al*. [34], But higher than other findings of previous studies [17,35,36] and the resistance rate of the combination amoxicillin and clavulanate is lower with 11.62%.

Tetracycline is a wide-spectrum bacteriostatic antibiotic indicated in cattle for the treatment of septicemia, respiratory, digestive, genitourinary, and interdigital infections; resistance to this antibiotic in *E. coli* is increasing [33,37], this resistance to tetracycline is mediated by plasmid, with a high variability of genetic determinants [38]. A large number of genetic determinants of tetracycline resistance allows sensitive bacteria to acquire resistance factors [39]. We reported a high resistance rate to tetracycline with 43.43%, which may be explained by its extensive use by clinical veterinarians in Algeria [30,40], this result is similar to the finding of Abbassi *et al*. [14] Other researchers have found lower rates of resistance to this molecule [17,39] as well as higher rates such as those obtained by Sawant *et al*. [34]; the high levels can be interpreted by the mechanisms of tetracycline resistance which are very ancient [41] and that tetracycline is a naturally occurring compound which bacteria can be exposed to in the outside in their use as human therapy, as prophylactics or as growth promoters in livestock [38].

Resistance to the many molecules of the cephalosporin family is often a result of stable mutations [38], a plasmid-mediated acquired resistance to the third-generation cephalosporins is now also reported by Payne and Amyes [42]. Bacteria can easily retain these stable mutations which confer resistance to cephalosporins, even in the absence of selective pressure to maintain resistance [39].

In our study, the first-generation cephalosporins are represented by cephalothin, for which a moderate resistance rate is observed 16.16%, which is in agreement with the results of Sayah *et al*. [39] The prevalence of resistance to the second- and third-generation cephalosporins, predominantly cefoxitin and cefotaxime, is low in our study with 2.02% and 4.54%, respectively, although nine *E. coli* strains ESBL producing were identified using a double-disk synergy test with cefotaxime and amoxicillin-clavulanate disks, other studies have reported the absence of cefotaxime resistance [14,28,35,43] and a similar resistance rate to our result for the third-generation cephalosporins is found [44].

In Algeria, several studies have been performed on the characterization of extended-spectrum ß-lactamases on many hosts, in humans by Touati *et al*. and Iabadene *et al*. [21,45], in poultry by Belmahdi *et al*. [23] and Meguenni *et al*. [24], and in pets (dogs and cats) by Yousfi *et al*. [46], but there are not any studies that investigate the topic of ESBL in commensal *E. coli* in cattle.

For trimethoprim/sulfamethoxazole, a moderate resistance rate is observed (15.15%); this agrees with Li *et al*. [44]. In combination of the two molecules: Trimethoprim/sulfamethoxazole is synergistic and produces a wide-spectrum bactericidal effect [33], the use of this antibiotic in animal feed, like its uncontrolled use in human medicine, can contribute for a long time to the development and transmission of genes encoding this marker of resistance [47]; sulfonamide resistance is widely prevalent and cross-resistance between sulfonamides is complete [38], so caution in the use of these antibiotics is required.

In Algeria, many antibiotics have been banned by ministerial decision since 2006, such as gentamycin, ciprofloxacin, chloramphenicol, and nitrofurantoin, despite these prohibition resistance levels have been recorded for these molecules with 1.1%,7.07%, 4.04%, and 2.52%, respectively, and the National Network for the Surveillance of the Resistance of Bacteria to Antibiotics is still recording significant levels of resistance to chloramphenicol (23.5%) and furans (18.3%) for *E. coli* strains, despite their prohibition since December 24, 2006 [48].

Other researchers from other countries have also reported chloramphenicol resistance in *E. coli* isolates from chickens and pigs in the absence of phenicol use for many years [49,50]. Coresistance of chloramphenicol with other unrelated compounds seems to be the probable explanation, as coresistance caused by the use of sulfonamides and streptomycin due to gene linkage has been reported [50-53].

Weak resistance rates are observed for nalidixic acid with 8.08% and ciprofloxacin with 7.07%, noting that ciprofloxacin is prohibited, which may be explained by the fact that fluoroquinolone resistance is linked to a chromosome mutation and the development of resistance to one agent causes cross-resistance to other fluoroquinolones [39].

Bywater *et al*. have abnormally reported a relatively high incidence of ciprofloxacin resistance in *Campylobacter* spp., despite the prohibition of its use, such observations as for chloramphenicol, explains at least some differences between antibiotic resistance and veterinary use of certain classes of antibiotics.

Kanamycin is a relatively old molecule, but its resistance rate is low 6.56%, explained by the low use of its family (aminoglycosides) by Algerian veterinary practitioners [30]. For gentamycin, we found a low resistance rate with 1.01%; this resistance explained by its illegal use because it is prohibited for use in Algeria.

Colistin is the only antibiotic that has not presented resistance (0%), which is in agreement with the results of de Jong *et al*. [35], who conducted this study in five European countries. Colistin is widely used for the treatment of colibacillary infections in animals but in human medicine has been excluded from therapeutic protocols due to its particular renal toxicity and became an antibiotic prescribed only for the treatment of serious human infections caused by bacteria resistant to any other antibiotics [54]. Colistin is also one of the most critical antibiotics, which the WHO has recently recommended to reduce in food-producing animals [55]

### Coresistances and MDR rate

Analysis of coresistance for the 198 *E. coli* isolates (Table-3) showed that 63 isolates (31.82%) were sensitive to all antibiotics used in the study, 42 isolates (21.21%) were resistant to one antibiotic, 43 isolates (21.72%) to two antibiotics, 24 isolates (12.12%) to three antibiotics, and 26 isolates (13.13%) for more than three antibiotics. Based on Kruskal–Wallis test results (non-parametric test), it can be estimated that there is a significant difference between the different coresistance rates with p=0.09023, thus showing a wide variety of phenotypes (Table-4).

**Table 3:** Coresistances of the 198 *E. coli* isolates from cattle.

Number of *E. coli* isolates	Number (n) and percentages (%) of isolates resistant to					Kruskal–Wallis *χ*^2^
	
	No agent	One agent	Two agents	Three agents	More than three agents	p-value

	n (%)	n (%)	n (%)	n (%)	n (%)	
198	63 (31.82)	42 (21.21)	43 (21.72)	24 (12.12)	26 (13.13)	0.09023

*E. coli*=*Escherichia coli*

**Table 4:** Kruskal–Wallis test.

	A	B
A	1	0.01219
B	0.01219	1

**Mann–Whitney pair wise comparison**		

H (K2)=6.818		
Hc (tie-corrected)=6.818		

On the other side, 45 isolates (22.73%) were MDR, which means resistant to three or more families of antibiotics since we considered beta-lactams and cephalosporins as two different families.

MDR bacteria present an impending risk to human and animal health, considering the limitations that they impose on the selection of antibiotic therapy for infections as well as the dangers of therapeutic failure. The MDR reported in our study can be the result of an independent resistance for each antibiotic or a coresistance.

A few major factors can contribute to increase bacterial MDR: The transfer of resistance determinants by movable genetic elements including plasmids, transposons, and gene cassettes into integrons and by changing regulation in mar locus [56].

Due to the indiskriminate exploitation of antimicrobial agents, high incidence of MDR may apparently occur, which may ultimately replace drug-sensitive microorganisms in the saturated antibiotic environment [7]. The MDR rate we have recorded is higher than that recorded by other authors [36,39,43], which motivates the monitoring of MDR *E. coli* strains.

### Variety of antimicrobial resistance phenotypes

Depending to their antibiotic resistance phenotypes, the 198 isolates of *E. coli* belong to 44 different phenotypes (Figure-2), thus showing a large variety of resistances, ranging from one antimicrobial to a combination of 10 (Table-5).

**Table 5:** Patterns of antimicrobial resistance phenotypes for *Escherichia coli* strains isolated in the study, with antibiogram pattern codes.

Number of resistances	Antibiogram patterns	Code of patterns	Number of strains
0	Susceptible to all antimicrobials	1	63
1	AMP	2	29
	KF	3	1
	FOX	4	1
	TE	5	10
	CIP	6	1
2	AMP+KF	7	6
	AMP+AMC	8	1
	AMP+TE	9	29
	AMP+NA	10	2
	K+TE	11	1
	TE+SXT	12	2
	TE+CIP	13	2
3	AMP+KF+AMC	14	1
	AMP+KF+SXT	15	1
	AMP+KF+TE	16	4
	AMP+KF+F	17	1
	AMP+AMC+SXT	18	2
	AMP+AMC+TE	19	1
	AMP+K + TE	20	3
	AMP+SXT+TE	21	11
4	AMP+KF+AMC+FOX	22	1
	AMP+KF+AMC+SXT	23	1
	AMP+KF+AMC+F	24	1
	AMP+AMC+TE+C	25	1
	AMP+K + SXT+TE	26	2
	AMP+K + TE+NA	27	1
5	AMP+KF+AMC+TE+CIP	28	1
	AMP+KF+AMC+TE+C	29	2
	AMP+KF+CTX+TE+NA	30	1
	AMP+SXT+TE+NA+CIP	31	2
6	AMP+KF+AMC+FOX+SXT+TE	32	1
	AMP+KF+AMC+CTX+TE+NA	33	1
	AMP+KF+AMC+TE+CIP+C	34	1
	AMP+AMC+K + SXT+TE+C	35	1
7	AMP+KF+AMC+CTX+TE+NA+CIP	36	1
8	AMP+KF+AMC+CTX+SXT+TE+NA+CIP	37	1
	AMP+KF+AMC+CTX+TE+NA+CIP+F	38	1
	AMP+KF+CTX+K + SXT+TE+NA+CIP	39	1
	AMP+KF+CTX+K + SXT+TE+NA+F	40	1
9	AMP+KF+AMC+FOX+SXT+TE+NA+CIP+F	41	1
	AMP+AMC+K + CN+SXT+TE+NA+CIP+C	42	1
10	AMP+KF+AMC+CTX+K + CN+SXT+TE+NA+C	43	1
	AMP+KF+AMC+CTX+K + SXT+TE+NA++CIP+C	44	1

AMP=Ampicillin, KF=Cephalothin, FOX=Cefoxitin, TE=Tetracycline, CIP=Ciprofloxacin, AMC=Amoxicillin+clavulanate, NA=Nalidixic acid, K=Kanamycin, SXT=Trimethoprim/sulfamethoxazole, F=Nitrofurantoin, CTX=Cefotaxime, C=Chloramphenicol, CN=Gentamycin

The two most frequent phenotypes with the same rate are AMP and AMP-TE with 14.65%, followed by the combination AMP-SXT-TE with 5.55% and TE with 5.05%. All MDR phenotypes are AMP resistant with a lower resistance level to tetracycline, which suggests that *E. coli* strains resistant to these antibiotics have an increased ability to be resistant for other antimicrobials, it has been reported in several previous studies that the most common phenotypes included a sole resistance to tetracycline or in association with other antibiotics [17,39,43].

There were nine different phenotypes detected with a rate of 4.54% producing extended-spectrum ß-lactamases and all are MDR ranging from five antimicrobials to a combination of 10. ESBL phenotype includes resistance to penicillins and cephalosporins, with the exception of cephamycins, which is the cause of many therapeutic failures [29], which requires the surveillance of strains with this type of phenotype.

The propagation of enterobacterial strains producing ESBL is a complex phenomenon involving three mechanisms. The first is clonal dissemination, where a strain producing ESBL can spread through horizontal contact between individuals. The second type is the transmission of one or several plasmids to another bacterial strain of the same or different species. The third is the transfer of resistance elements present in transposons or integrons between different plasmids. Plasmids often have other resistance genes (including aminoglycosides, tetracyclines, sulfonamides, and trimethoprim), hence, the notion of coresistance, coexpression, and coselection [57].

In this study, the high variability of resistance phenotypes can be explained by coresistance (acquisition of resistance to several antibiotics of different classes), as the plasmids exchanged usually have several resistance genes such as the coresistance of *E. coli* to cephalosporins, penicillins, chloramphenicol, tetracyclines, and fluoroquinolones.

In the same way as for cross-selection, the use of an antibiotic to which the bacterium resists will allow the coselection of all the resistances supported by the same plasmid. The diffusion and acquisition of resistances are independent of the use of antibiotics, but their use, without being aware of the state of bacterial sensitivity, can quickly cause the selection of MDR strains [58].

- The upper margin indicates the antibiogram patterns of phenotypic antimicrobial resistance detected from 1 to 44
- The left margin indicates the distance between the different clusters.

## Conclusion

Antibiotic sensitivity of 198 isolates of *E. coli* collected from cattle in Eastern Algeria showed high frequencies of resistance to AMP and TE and a high level of *E. coli* producing ESBLs was detected. ESBL strains are the cause of many therapeutic failures, which require the surveillance of strains that contain this phenotype. The coresistance analysis showed a high rate of MDR strains, 44 different phenotypes were also detected, showing a high variety of resistance, ranging from 1 to 10 antimicrobials combination.

The high rate of antimicrobial resistance in commensal *E. coli* from Algerian livestock emphasizes the urgency of intervention to implement the measures to control the use of antimicrobials, in particular, by private veterinarians, and to strengthen networks to control bacterial resistance to antibiotics, which can spread to humans and the environment.

## Authors’ Contributions

DB collected the samples and provided the bacteriological analyzes. AB contributed by statistical analyses and the creation of the map; DB and AB prepared the manuscript; and NB supervised the manuscript. All authors read and approved the final manuscript.
